# Mechanism of central hypopnoea induced by organic phosphorus poisoning

**DOI:** 10.1038/s41598-020-73003-5

**Published:** 2020-09-28

**Authors:** Kazuhito Nomura, Eichi Narimatsu, Hiroyuki Inoue, Ryoko Kyan, Keigo Sawamoto, Shuji Uemura, Ryuichiro Kakizaki, Keisuke Harada

**Affiliations:** Department of Emergency Medicine, Sapporo Medical University Hospital, Minami1-jo nishi 16 Chome 291, Chuo-ku, Sapporo, Hokkaido 060-8543 Japan

**Keywords:** Biological techniques, Electrophysiology, Extracellular recording, Physiology, Neurophysiology, Cellular neuroscience, Neural circuits, Excitability, Inhibition, Inhibition-excitation balance, Action potential generation, Neuroscience, Neuronal physiology, Intrinsic excitability, Neurological disorders, Neurotoxicity syndromes

## Abstract

Whether central apnoea or hypopnoea can be induced by organophosphorus poisoning remains unknown to date. By using the acute brainstem slice method and multi-electrode array system, we established a paraoxon (a typical acetylcholinesterase inhibitor) poisoning model to investigate the time-dependent changes in respiratory burst amplitudes of the pre-Bötzinger complex (respiratory rhythm generator). We then determined whether pralidoxime or atropine, which are antidotes of paraoxon, could counteract the effects of paraoxon. Herein, we showed that paraoxon significantly decreased the respiratory burst amplitude of the pre-Bötzinger complex (*p* < 0.05). Moreover, pralidoxime and atropine could suppress the decrease in amplitude by paraoxon (*p* < 0.05). Paraoxon directly impaired the pre-Bötzinger complex, and the findings implied that this impairment caused central apnoea or hypopnoea. Pralidoxime and atropine could therapeutically attenuate the impairment. This study is the first to prove the usefulness of the multi-electrode array method for electrophysiological and toxicological studies in the mammalian brainstem.

## Introduction

The pre-Bötzinger complex (preBötC) in the ventrolateral lower brainstem is essential for the formation of the unconscious breathing rhythm in mammals^1,2^. This is because the cyclic burst excitation generated from preBötC synchronizes with the respiratory rhythm through phrenic nerve firing and the diaphragmatic contractions, and destruction of preBötC causes the disappearance of the rhythm. Periodic respiratory burst excitation has also been confirmed from an island specimen derived by isolating preBötC in an island shape to block input from other neurons^2^. PreBötC is thus considered the core and origin of respiratory rhythm formation. Although numerous studies on preBötC and the related regions were previously conducted, in vitro experiments using thin slices (i.e. respiratory slices) containing the preBötC region are most appropriate to discuss the pharmacological responses limited to the preBötC region. The preBötC receives ascending or descending signals from different regions^3–5^ and causes rhythm variation according to the signals. However, respiratory slices can block these signal inputs, thereby enabling the verification of preBötC behaviour alone. Respiratory rhythm abnormalities are often observed with organophosphorus cholinesterase inhibitor poisoning^3,6–9^. Peripheral and central mechanisms are involved in this phenomenon. However, the central mechanism develops in the early stage and is a highly lethal pathological condition accompanied by severe consciousness disorder^8,9^. In some in vivo studies with rodents, central apnoea or hypopnoea was reported to occur when organophosphorus drugs were administered within or nearby the preBötC region^7,10,11^. Further, they were previously confirmed that organophosphorus drugs cause central apnoea or hypopnoea. Although respiratory motion, which involves the reduction in respiratory rate and tidal volume, was evaluated in those studies, the changes in preBötC electrical activity have yet to be reported. In a similar study, a reversible cholinesterase inhibitor, physostigmine, was administered to respiratory slices and the changes in preBötC electrical activity were observed. According to the study results, the activity of preBötC was generally increased by physostigmine administration^12^. Because respiratory exercise should be activated in response to increased preBötC activity, the findings of this in vitro study^12^ do not agree with those of the above-mentioned in vivo study^6,7,10,11^. Further, as there are no study reports that clarify this inconsistency, the pathology of central apnoea or hypopnoea by organophosphorus cholinesterase inhibitors remains unknown. To resolve this inconsistency and elucidate the mechanism of central apnoea or hypopnoea induced by organophosphorus drugs, we conducted an in vitro neuro-electrophysiological experiment using respiratory slices from juvenile rats.

## Results

**Figure 1:**
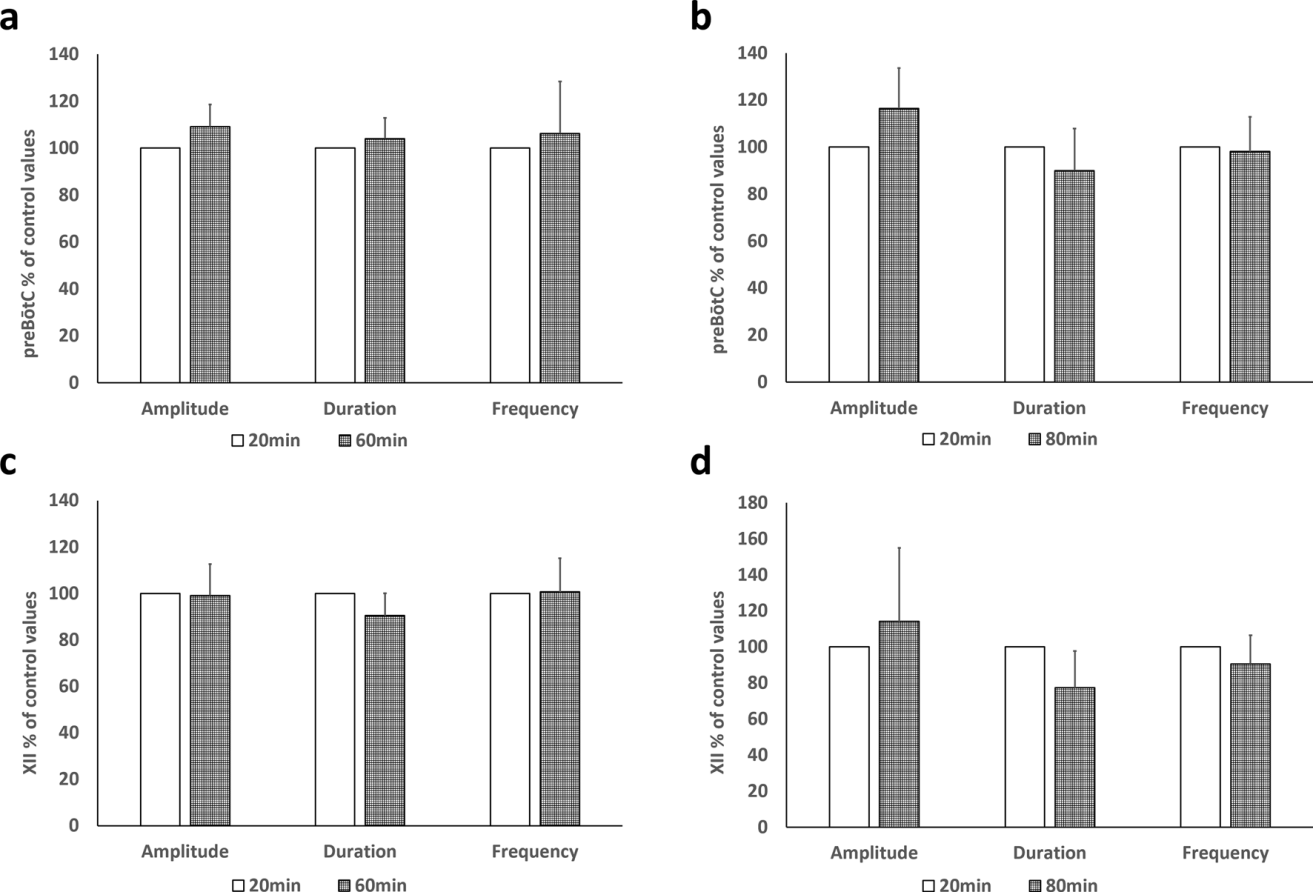
Results of the control experiments. (**a**,**c**) rACSF was perfused for 60 min. The 1-min mean for each item was calculated at the 20th and 60th min. The value at the 60th min relative to that at the 20th min was expressed as a ratio. No statistically significant difference was found by the paired *t* test. preBötC: amplitude, *p* = 0.083; duration, *p* = 0.36; and frequency, *p* = 0.56; XII: amplitude, *p* = 0.89; duration, *p* = 0.078; and frequency, *p* = 0.92). (**a**) Graph for preBötC, (**c**) graph for XII. (**b**,**d**) rACSF was perfused for 80 min and the 1-min mean for each item was calculated at the 20th and 80th min. The value at the 80th min relative to that at the 20th min was expressed as a ratio. No statistically significant difference was found by the paired *t* test. (preBötC: amplitude, *p* = 0.095; duration, *p* = 0.25; and frequency, *p* = 0.78; XII: amplitude, *p* = 0.47; duration, *p* = 0.056; and frequency, *p* = 0.24). (**b**) Graph for preBötC, (**d**) graph for XII. *preBötC* pre-Bötzinger complex, *XII* hypoglossal nucleus.

In the groups respectively administered recording artificial cerebrospinal fluid (rACSF) alone for 60 min (n = 6) and 80 min (n = 6), a significant difference in the endpoints (burst amplitude, burst frequency, and burst duration) was not found when the control value obtained at the 20th min was compared to the values obtained at the 60th and 80th min (Fig. 1). The 3rd phase for measuring the maximum effective values was shifted by 20 min between the simultaneous treatment group and the pre/post-treatment group (see protocol section). We performed multiple comparisons for amplitude, duration, and frequency among the 3 groups: control value (20th min), 40th min, and 60th min. However, no significant difference was observed.

### Effects of Paraoxon (Figs. 2, 3, Table 1)

**Figure 2:**
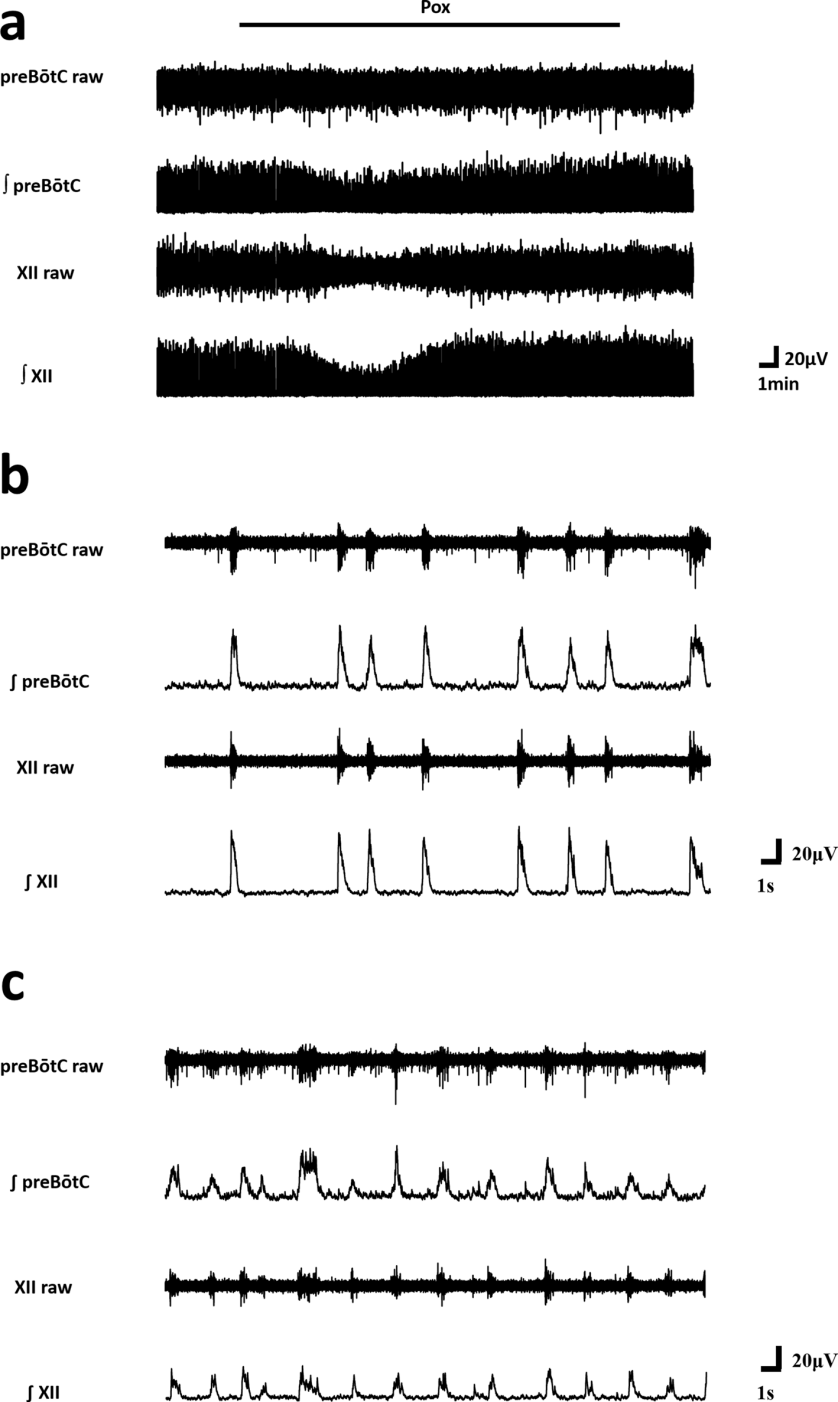
Changes in waveform after the administration of paraoxon. (**a**) An example of respiratory bursts in the preBötC and XII around the time of paraoxon administration. The lower subrows show the integrated bursts. (**b**) An enlarged drawing of the respiratory bursts in the preBötC and XII at the control values. (**c**) An enlarged drawing of the respiratory bursts in the preBötC and XII at the time of paraoxon administration. ∫*preBötC* integrated row for the preBötC, ∫*XII* integrated row for XII.

**Figure 3:**
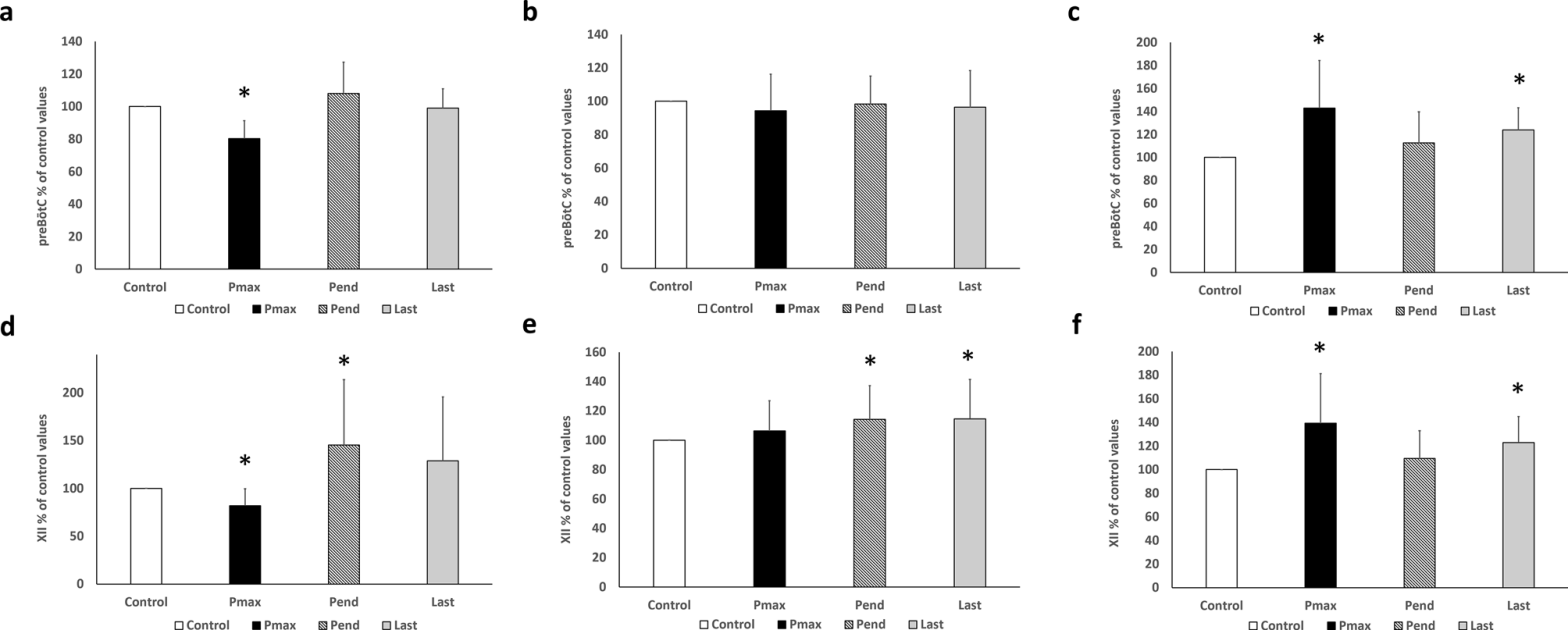
Effects of paraoxon administration. The changes at each endpoint over time as a ratio to the control value for the Pox alone group. (**a**) Graph for preBötC amplitude, (**b**) graph for preBötC duration, (**c**) graph for preBötC frequency, (**d**) graph for XII amplitude, (**e**) graph for XII duration, (**f**) graph for XII frequency. Control: the 1-min mean of each item at the 20th min in the 2nd phase (Table 1) was calculated and defined as the control value. The control value was 100% when the ratio was calculated according to the change over time. Pmax: the values for the maximum effect were defined as burst amplitude, duration, and frequency. The most decline in mean burst amplitude per min occurred at 20 min of paraoxon administration (3rd phase in Table 1). This value was expressed as a ratio relative to the control value. Pend: Paraoxon was perfused for 20 min. The 1-min mean of each endpoint at the 20th minute (the last 1 min of the 3rd phase in Table 1) was calculated and expressed as a ratio relative to the control value. Last: rACSF was perfused for 20 min because of wash out (4th phase in Table 1). The mean of each parameter per min at the 20th min was calculated and expressed as a ratio relative to the control value. *Statistically significant differences by one-way ANOVA and Bonferroni analysis (*p* < 0.05).

**Table 1:** Summary of the study design used for drug application.

Drug application protocol				
Group	1st phase (20 min)	2nd phase (20 min)	3rd phase (20 min)	4th phase (20 min)
Pox alone				
Application of Pox		rACSF	Pox	Wash out
PAM and Pox				
Pre-treatment with PAM	rACSF	PAM	PAM + Pox	Wash out
Simultaneous treatment with PAM		rACSF	PAM + Pox	Wash out
Post-treatment with PAM	rACSF	Pox	PAM + Pox	Wash out
ATR and Pox				
Pre-treatment with ATR	rACSF	ATR	ATR + Pox	Wash out
Simultaneous treatment with ATR		rACSF	ATR + Pox	Wash out
Post-treatment with ATR	rACSF	Pox	ATR + Pox	ATR + Pox
Physo alone				
Application of Physo		rACSF	Physo	Wash out

*rACSF* recording artificial cerebrospinal fluid warmed to 30 °C, *Pox* paraoxon (10 μM, an acetylcholinesterase inhibitor), *PAM* Pralidoxime (100 μM, an oxime), *ATR* Atropine (1 μM, a non-selective acetylcholine receptor antagonist), *Physo* Physostigmine (100 μM, an acetylcholinesterase inhibitor), Drugs were administered with superfusing rACSF. Wash out was performed with rACSF.

Paraoxon (Pox) is a typical and irreversible acetylcholinesterase inhibitor. A total of 30 rats were employed in the experiment with the Pox alone group. The dose concentration of Pox was 10 μM. The burst amplitude of preBötC significantly decreased at the maximum effective value (Pmax: 80.3 ± 11.0%, *p* < 0.05), however, there was no difference relative to the control in Pend and Last (Figs. 2, 3a). Additionally, the burst amplitude of hypoglossal nucleus (XII) significantly decreased at the maximum effective value (Pmax: 82.0 ± 17.6%, *p* < 0.05), but significantly increased in the end of 3rd phase in Table 1 (Pend: 145.2 ± 68.4%, *p* < 0.05) (Figs. 2, 3d). No difference was identified relative to the control value in the end of 4th phase in Table 1 (last in Fig. 3d). Burst duration did not show any significant difference in preBötC throughout the process (Fig. 3b); however, a significant increase with XII in Pend (114.1 ± 23.0%, *p* < 0.05) was found as well as an increase in Last (114.6 ± 26.9%, *p* < 0.05) (Fig. 3e). Burst frequency significantly increased with the maximum effective value (Pmax in Fig. 3c,f) in both preBötC and XII (preBötC: 143.0 ± 41.3%, *p* < 0.05; XII: 139.2 ± 42.0%, *p* < 0.05) and increased in the end of 4th phase (last in Fig. 3c,f) (preBötC: 123.8 ± 19.4%, *p* < 0.05; XII: 122.8 ± 22.0%, *p* < 0.05).

### Effects of Pralidoxime (Fig. 4, Table 1)

**Figure 4:**
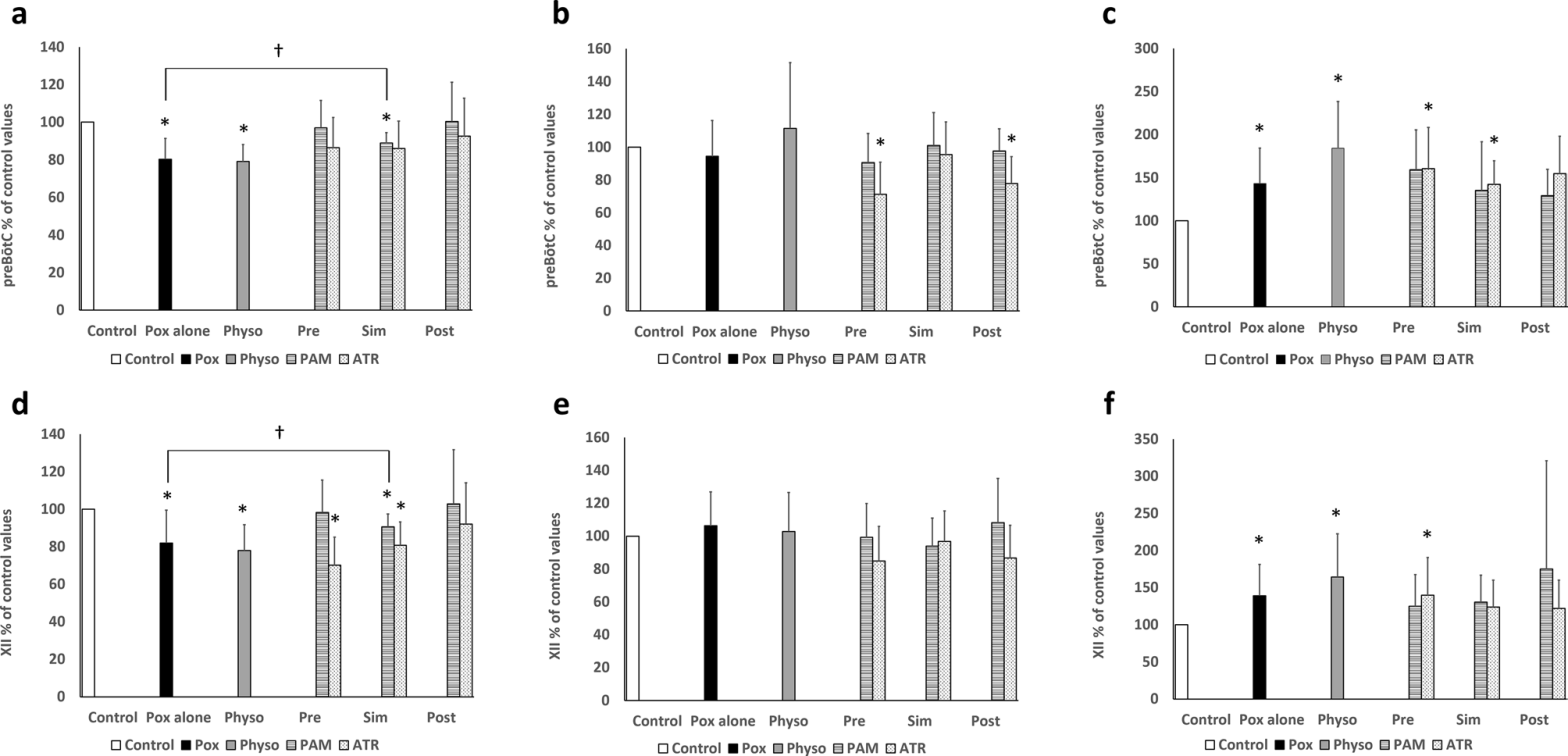
Results related to therapeutic intervention and physostigmine administration. The results of the value for the maximum effect of each item relative to that of the control as a ratio for the 4 groups presented in Table 1. (**a**) Graph for preBötC amplitude, (**b**) graph for preBötC duration, (**c**) graph for preBötC frequency, (**d**) graph for XII amplitude, (**e**) graph for XII duration, (**f**) graph for XII frequency. Control: the 1-min mean of each item at the 20th min in the 1st or 2nd phase (Table 1) was calculated for each group and defined as the control value. The control value was 100% when the ratio was calculated according to the change over time. Pox alone: the value for the maximum effect in the 3rd phase (Table 1) in the Pox alone group was expressed as a ratio relative to the control value. Physo: The value for the maximum effect in the 3rd phase (Table 1) of physostigmine administration was expressed as a ratio relative to the control value. Pre: The value for the maximum effect in the 3rd phase (Table 1) for the pre-treatment group was expressed as a ratio relative to the control value. Sim: The value for the maximum effect value in the 3rd phase (Table 1) for the simultaneous treatment group was expressed as a ratio relative to the control value. Post: The value for the maximum effect in the 3rd phase (Table 1) in the post-treatment group was expressed as a ratio relative to the control value. *Statistically significant differences by one-way ANOVA and Bonferroni analysis (*p* < 0.05). ^†^Statistically significant differences by *F* test and unpaired *t* test (*p* < 0.05).

Pralidoxime (PAM) is an oxime and representative antidote against organophosphorus drugs. A total of 10 rats were used in the pre-treatment group experiment. Regarding the maximum effective value when Pox (10 μM) and PAM (100 μM) were administered (3rd phase in Table 1), there was no significant difference in burst amplitude (Fig. 4a,d), burst Duration (Fig. 4b,e), or burst frequency (Fig. 4c,f) for preBötC and XII compared to the control.

A total of 12 rats were used in the simultaneous treatment experiment. With regard to the maximum effective value when Pox (10 μM) and PAM (100 μM) were administered simultaneously (3rd phase in Table 1), the burst amplitudes of preBötC and XII decreased significantly (preBötC: 88.9 ± 5.5%, *p* < 0.05; XII: 90.6 ± 7.0%, *p* < 0.05). However, the amplitude was significantly higher in the simultaneous treatment group than the maximum effective value of Pox alone, as evidenced by the F test and unpaired *t* test (preBötC: 80.3 ± 11.0% vs 88.9 ± 5.5%, *p* < 0.05; XII: 82.0 ± 17.6% vs 90.6 ± 7.0%, *p* < 0.05) (Fig. 4a,d). There was no significant difference in the burst duration (Fig. 4b,e) or the frequency (Fig. 4c,f) of preBötC and XII compared to the control.

A total of 10 rats were used in the post-treatment group experiment. Regarding the maximum effective value when Pox (10 μM) and PAM (100 μM) were administered (3rd phase in Table 1), there was no significant difference between burst amplitude, duration, and frequency for preBötC and XII relative to the control values.

### Effects of Atropine (Fig. 4, Table 1)

A total of 11 rats were used in the pre-treatment group experiment. Regarding the maximum effective value when Pox (10 μM) and atropine (1 μM) were simultaneously administered (3rd phase in Table 1), burst amplitude did not differ from the control in preBötC (Fig. 4a); however, a significant decrease in XII (70.2 ± 15.0%, *p* < 0.05) (Fig. 4d) was observed relative to the control. Compared to the Pox alone group by the F test and unpaired *t* test, no significant difference was observed. A significant difference in burst duration was observed for preBötC (71.2 ± 19.6%, *p* < 0.05) (Fig. 4b) but not XII (Fig. 4e). There was a significant difference in burst frequency for both preBötC and XII (preBötC: 160.5 ± 47.8%, *p* < 0.05; XII: 139.7 ± 50.9%, *p* < 0.05) (Fig. 4c,f).

A total of 9 rats were used in the simultaneous treatment experiment. Regarding the maximum effective value, when Pox (10 μM) and atropine (1 μM) were simultaneously administered (3rd phase in Table 1), there was no difference in burst amplitude between preBötC and control (Fig. 4a); however, a significant difference relative to XII (80.8 ± 12.3%, *p* < 0.05) was found (Fig. 4d). Based on the *F* test and unpaired *t* test, no significant difference relative to the Pox alone group was found. There was no significant difference in burst duration in both preBötC and XII (Fig. 4b,e). Additionally, burst frequency was significantly different for preBötC (142.1 ± 27.2%, *p* < 0.05) (Fig. 4c) but not for XII relative to the control (Fig. 4f).

A total of 11 rats were used in the post-treatment group experiment. Regarding the maximum effective value when Pox (10 μM) and atropine (1 μM) were simultaneously administered (3rd phase in Table 1), there was no difference in burst amplitude or frequency relative to the control values in both preBötC and XII, except for the duration of preBötC (77.8 ± 16.4%, *p* < 0.05) (Fig. 4b).

### Effects of physostigmine (Fig. 4, Table 1)

A total of 9 rats were used in the experiment for the group administered physostigmine alone (3rd phase in Table 1). When the maximum effect of physostigmine (100 μM) was exerted, the burst amplitude of preBötC and XII decreased significantly (preBötC: 79.1 ± 9.1%, *p* < 0.05; XII: 78.0 ± 13.8%, *p* < 0.05) (Fig. 4a,d). Both preBötC and XII exhibited no difference in burst duration relative to the control. The burst frequency significantly increased in preBötC and XII (preBötC: 184.1 ± 54.3%, *p* < 0.05; XII: 164.2 ± 58.3%, *p* < 0.05) (Fig. 4c,f).

## Discussion

The multi-electrode arrays method (MEA method) is widely used in electrophysiology studies involving the hippocampus, retina, myocardium, and cultured cells^13,14^; however, a prior report of its use with the respiratory centre has not been published. The MEA method has the following advantages: (1) It does not require a Faraday cage because it is insulated from the influence of external electrical noise. (2) If researchers just apply drugs in the perfusion solution of the MEA system, they can perform pharmacological experiments. (3) High flow rate perfusion of solutions often moves electrodes (e.g. needle electrodes) and causes experimental failure when using the classical method. Meanwhile, it is easier to perform experiments successfully because the MEA electrodes are immovable. (4) In the classical method, electrodes inserted into the respiratory slice cause tissue damage. When using the MEA method, only close attachment of the slice and electrodes is needed. The MEA method causes minimal tissue damage and can provide data from a relatively healthy state. We not only established a new experimental method using MEA but also demonstrated for the first time its usability for studies in the brainstem region.

There are many exclusive MEA products for various tissues, as shown above. They are designed in compliance with the tissue structure (e.g. distance of target sites) and tissue characteristics (e.g. the rounded surface of the retina). They have optimized the numbers, materials, shapes, sizes, and layouts of electrodes to simply measure local field potentials. Exclusive MEA products can measure multiple local field potentials simultaneously in corresponding tissues. Unfortunately, however, an exclusive MEA for the respiratory slice does not exist yet. We used a 60EcoMEA-gr-12 mm because it is the most popular and low-cost option. It permits us to measure local field potentials in only the ipsilateral preBötC/XII simultaneously because of the electrode layout. However, if a new special MEA for the respiratory slice is created, it will permit us to more simply measure local field potentials at the same time in multiple target sites (e.g. bilateral preBötC/XII/hypoglossal nerve).

Based on the in vivo findings^3,6,7,10,11^, we hypothesized that organophosphorus cholinesterase inhibitors directly impair preBötC. As the administration of Pox was found to significantly reduce the burst amplitude of preBötC, this is the first electrophysiological proof that Pox impairs preBötC activity. We also applied PAM and atropine to a Pox intoxication model and confirmed that they could inhibit the reduction in the preBötC burst amplitude induced by Pox. Such finding demonstrates that PAM and atropine are therapeutically effective for treating the preBötC impairment caused by Pox. Pox is a potent acetylcholinesterase inhibitor that exerts toxicity by binding to the esterified degradation site of acetylcholinesterase to deactivate acetylcholinesterase and create acetylcholine overload^15^. In the muscle tissue of patients with Pox poisoning, muscle fibres are excessively excited due to an extremely high concentration of acetylcholine at the neuromuscular junction. As a result, muscle contraction (fasciculation) continues in a convulsive manner, resulting in hypertonic paralysis. We hypothesized that Pox reaching the brainstem creates acetylcholine overload in the respiratory centre, which causes excitotoxicity and results in functional impairment of preBötC. As a result, a significant decrease in the amplitude of preBötC was confirmed by the administration of Pox, demonstrating that Pox directly impairs preBötC (Figs. 2,3). In a similar study, a reversible acetylcholinesterase inhibitor, physostigmine, which differs from Pox, was administered to respiratory slices, and the results showed that physostigmine enhanced the overall activity of preBötC^12^. However, this result was observed since the increase in acetylcholine concentration was within the physiological range and was not aimed at poisoning concentration, as done in our study.

The unknown toxicity specific to Pox, which is not present in physostigmine, might be the cause of this finding. As a result, we administered physostigmine at a level 10 times greater than that in the previous study. Based on our findings, preBötC burst amplitude decreased and burst frequency increased with physostigmine in a similar manner to that with Pox. In conclusion, if the concentration of acetylcholine through acetylcholinesterase inhibition reaches the toxic range for both Pox and physostigmine, acetylcholine exhibits excitotoxicity and directly impairs preBötC. Some previous study revealed that as the acetylcholine level increases in the brainstem tissue, preBötC excitation becomes high, burst frequency increases, and burst duration deviates from the inspiratory phase and extends to the expiratory phase, ultimately attenuating synaptic binding^12,16^. This finding is consistent with the increased burst frequency caused by Pox administration. PreBötC was also recognized to be formed with several groups of interneurons. Because the neurons are communicating at synapses, they are characterized by cyclic synchronous burst excitation. The attenuation of synaptic connectivity indicates the loss of synchronization between neurons. As a result, a burst with a large amplitude is not generated when the excitation spreads throughout the preBötC, as observed in the healthy state.

Because Pox is an irreversible acetylcholinesterase inhibitor, the acetylcholine overload induced by Pox should be maintained. Prior to study initiation, we predicted that the inhibitory effect of preBötC would continue after wash out. In fact, there was an increase in frequency after the end of Pox administration (Fig. 3c). However, the amplitude spontaneously recovered during Pox administration and no difference was found relative to the control values. There is a possibility that desensitization of acetylcholine receptors contributes to the spontaneous recovery (Figs. 2, 3a). Application of physostigmine increases the amplitude, frequency, and duration of the respiratory burst but additional application of 4-diphenylacetoxy-*N*-methylpiperidine (4-DAMP, M3 muscarinic acetylcholine receptor-selective antagonist) was shown to block the increase in amplitude in a previous study^12^. Therefore, it is likely that M3 desensitization resulted in the recovery of the amplitude of preBötC by the reducing excessive excitement of preBötC in the 3rd phase of Pox alone.

There are two subtypes of nicotinic receptors in inspiratory neurons, α4β2 and α7; both receptors bind acetylcholine and excite inspiratory neurons^12,17^. The application of highly concentrated nicotine-induced respiratory arrest in an earlier study^18^. Furthermore, another past study showed higher agonist concentration induces rapid α7-nicotinic acetylcholine receptor desensitization^19^. Nicotinic receptors might be relevant to Pox-induced central hypopnoea and the recovery from the decreasing preBötC amplitude. Additional studies with Pox and receptor antagonists, e.g. methyllycaconitine (selective α7-nicotinic receptor antagonist), are needed to confirm this hypothesis. A previous study has shown that Pox influences different transmission pathways, including the promotion of glutamate release from the presynaptic membrane and further inhibition of GABA uptake^20^. Alterations in the metabolic pathways related to excitation transmission within neurons were also recognized. This unexpected result may be due to a series of changes; however, because this study was performed with extracellular potential records alone, the detailed mechanisms between synapses and within cells are unknown. More detailed studies on the intracellular environment, such as studies with patch-clamp techniques, are thus required.

PAM and atropine were employed in the therapeutic intervention experiments to demonstrate that the Pox-induced impairment of preBötC was caused by acetylcholine overload. PAM is known to restore acetylcholinesterase activity by breaking the bond between Pox and acetylcholinesterase, thereby causing the degradation of excessive acetylcholine and normalization of neurotransmission^15^. Precedent application of PAM (pre-treatment with PAM) demonstrates a more effective inhibitory action on the binding of acetylcholinesterase and Pox; therefore, excessive excitement of neurons caused by acetylcholine overload is more greatly reduced than with simultaneous treatment with PAM, as has been performed in past studies^21,22^. PAM has greater efficacy in pre-treatment than in simultaneous treatment (Fig. 4a,d). This finding in our study is in agreement with that of past studies. As shown in Fig. 3a, the reduced amplitude of preBötC recovered spontaneously until termination of Pox application (Pend). In post-treatment with PAM, application of Pox alone was started from the 2nd phase, whereas additional PAM application was started from the 3rd phase (Table 1). In brief, it is difficult to determine whether PAM contributes to the recovery of preBötC amplitude in the 3rd phase of post-treatment with PAM because spontaneous recovery of the preBötC amplitude occurred until the 3rd phase (Table 1, Figs. 3a, 4a).

Atropine is a non-specific muscarinic receptor blocker that inhibits excessive excitation of neurons by inhibiting the binding of acetylcholine to muscarinic receptors when an excessive increase in acetylcholine occurs between synapses^12,21,23^. In addition, the administration of atropine attenuated the decrease in preBötC amplitude, demonstrating that excessive acetylcholine impairs preBötC. As shown in Fig. 4a, atropine (1 μM) suppressed Pox-induced preBötC amplitude depression. A previous study addressed that 4-DAMP antagonizes the acetylcholine-induced increase in duration, and the anti-M3 effect of atropine decreases duration^12^. In Fig. 4b, the decreased duration of preBötC in the atropine treatment group implies a side effect of atropine. This study focused on antagonistic effect of atropine on Pox-induced amplitude depression; therefore, we set the concentration of atropine at 1 μM to exert an antagonistic effect. Several inconsistencies were identified between preBötC and XII, except for the significant decrease in burst amplitude after Pox administration. For example, treatment with atropine suppressed the preBötC burst amplitude reduction induced by Pox, but not that of XII (Fig. 4a,d). The anticholinergic effects of atropine may thus be involved or may indicate that neurons in preBötC and XII exhibit different responses to atropine.

In previous studies, the authors conducted in vivo or en bloc (brainstem-spinal cord preparation) experiments. They revealed nothing more than the rough pathological mechanism in a wide area of the respiratory centre and did not elucidate electrophysiological changes in preBötC/XII. The physiological function or regulation of the respiratory centre has not been completely elucidated as they are incredibly complicated. Therefore, first of all, it is necessary to elucidate the mechanism of central apnoea or hypopnoea induced by organophosphorus drugs in studies that focus precisely on preBötC because preBötC seems to be the kernel for respiratory rhythm generation and one of culprit lesions in central apnoea or hypopnoea. The present study was carried out in vitro with respiratory slices to clarify Pox-induced direct impairment of the narrow area involving the preBötC. Fleming et al. suggested in their study in an anaesthetized cat that neostigmine suppressed the respiratory centre indirectly by altering afferent inputs and consequently phrenic nerve activity^3^. Their findings suggest that the acetylcholinesterase inhibitor-induced central apnoea or hypopnoea might be caused by a mechanism other than direct preBötC impairment. For example, the Kölliker-Fuse nucleus in the pons or the spinal neural circuit, affect preBötC behaviour^5^. Meanwhile, the respiratory slice is independent of them and exhibits only the autonomous rhythmic bursting of preBötC. In this study with the respiratory slice, we revealed that preBötC is just impaired by Pox, an organophosphorus drug, directly causing a culprit lesion for hypopnoea. Of course, the mechanism of central apnoea or hypopnoea is not completely elucidated, but our findings are highly significant to provide further understanding in the future and contribute further evidence that preBötC is also a target of treatment. For these reasons, this study has great originality and importance. This requires further analysis in the future.

## Conclusion

Based on the findings presented herein, central apnoea or hypopnoea induced by organophosphorus acetylcholinesterase inhibitors was caused by the direct impairment of preBötC. PAM and atropine can be administered as antidotes for Pox. The MEA was demonstrated to be a useful method for in vitro system electrophysiology and pharmacology studies in the brainstem respiratory centre.

## Methods

### Slice preparation

The study began after the study protocol was approved by the Sapporo Medical University Institutional Animal Care and Use Committee. All experiments were conducted in accordance with the Regulations for the Management of Laboratory Animals at Sapporo Medical University and relevant guidelines. The experiment was performed with 114 SD newborn rats (age, 0–6 days-old). First, a low-temperature environment (0–4 °C) was produced using a cup of ice. Rats were exposed to ice-cold air in the cup to induce a deep hypothermic state. Because the thermoregulatory ability of neonatal rats is immature, deep hypothermia is easily induced and leads to a deep anaesthesia leaving rats unresponsive to painful stimuli^24^. After rats were deemed unresponsive to pinch stimuli, their forehead and supradiaphragmatic thorax were transected. Subsequently, the brainstem and spinal cord were rapidly isolated as a lump at 0–4 °C in sucrose-based artificial cerebrospinal fluid (sucrose 260 mM, KCl 2.5 mM, CaCl_2_ 0.5 mM, MgSO_4_ 10 mM, NaH_2_PO_4_ 1.25 mM, NaHCO_3_ 25 mM, glucose 25 mM, pH 7.4, 385 mOsm/L calculated value) saturated with 95% O_2_ and 5% CO_2_. The sucrose-based ACSF is helpful in making a ‘healthier slice’ and is widely used^25^. The anatomical drawing of neonatal rats and previous studies^1,12,26,27^ were employed to prepare the slices. From the obex region, transection was performed toward the rostral side with a slicer (NLS-MT; DOSAKA, Osaka, Japan) at a thickness of 400–600 μm (the rostral side was the inferior border of the 4th ventricle) to ensure the XII and the inferior olive nucleus were evident. Thereafter, the acute brainstem slices were prepared. Only one slice was prepared from each rat. The prepared slices were incubated for 20 min in rACSF (NaCl 123 mM, KCL 12 mM, CaCl_2_ 2.5 mM, MgSO_4_ 1.2 mM, NaH_2_PO_4_ 1.2 mM, NaHCO_3_ 25 mM, Glucose, 30 mM, pH 7.4, 383 mOsm/L, calculated value, 20–25 °C) with continuous bubbling of 95% O_2_ and 5% CO_2_, and transferred to the MEA chamber for recording.

### Recording and analysis

The positions of PreBötC and XII in the slice were identified using an inverted microscope; the slice was then moved to adhere preBötC/XII to the MEA arrays. The respiratory burst excitation of preBötC was transmitted to XII. As a result, a similar rhythmic burst synchronously appeared in preBötC and XII. If this similar synchronous rhythmic burst was confirmed in both XII and preBötC, it could serve as the rationale for respiratory burst^1,2^. To prove that the rhythmic burst of preBötC was indeed the respiratory burst, simultaneously and constantly recording both potentials of preBötC and XII were critical. The rostral side of the slice preparation was placed on the base of the MEA dish (60EcoMEA-gr-12 mm; Multi Channel Systems) or the recording electrode side. One side of the preBötC and the same side of XII were closely attached to the recording electrode. Gold was used as the electrode material: 100 μm, diameter; 30 kΩ, resistance; and 60 electrodes with an interval of 700 μm, arranged in an 8 × 8 grid fashion. The rACSF maintained at 30 °C with a warming device (95% O_2_ and 5% CO_2_) was perfused in the MEA chamber at a rate of 10–20 mL/h to immerse the slices in the ACSF. The MEA device (MEA1060; Multi Channel Systems, Reutlingen, Germany) was connected to an AD converter (PowerLab 16/30; ADInstruments Bella Vista, Australia) to measure and record the neuronal activity. The recorded neuronal activity was monitored on a computer in real time using an analysis software attached to PowerLab 16/30 (LabChart pro ver 8.1). The measurement data were recorded on a computer and analysed. The following three measurement items were employed: burst amplitude, duration and frequency. All bursts were integrated with a time constant of 0.05 s using LabChart for analysis. Burst frequency was directly measured as the number of bursts per minute. To derive the burst amplitude, the heights from baseline to the peaks of the integrated burst waveform were measured. To determine the burst duration, the time required for the burst to increase and return to baseline was measured. The mean burst amplitude and burst duration were calculated every min, and the ratio was calculated according to a control value of 100%.

### Drugs

Paraoxon (*O*,*O*-diethyl O-(4-nitrophenyl) phosphate, organophosphorus acetylcholinesterase inhibitor), PAM (2-pyridine aldoxime methiodide, oxime compound), and atropine (muscarinic receptor nonspecific antagonist) were obtained from Sigma-Aldrich. Because Pox is lipophilic, it was dissolved with dimethyl sulfoxide to achieve solubility in water. Thereafter, it was added to standard ACSF. The concentration of dimethyl sulfoxide in ACSF was adjusted to less than 0.1%.

### Protocol

The protocol (Table 1) was derived according to a previous study by the coauthors^21,28^. Briefly, rACSF was perfused at a high flow rate of 10–20 mL/min using drip infusion sets and a suction pump. The preBötC and XII were confirmed to display regular synchronous burst excitation before recording. The drugs were administered after rACSF was perfused for 20 min from the start of recording. Drug administration was carried out via the addition of the study drugs to rACSF and perfusion for 20 min. After completion of drug administration, rACSF was perfused for an additional 20 min to complete the procedure. In this study, we examined (1) Pox alone, (2) pre-, simultaneous, post-treatment with PAM or atropine, and (3) physostigmine alone.

### Statistics

The results are expressed as mean ± standard deviation. The mean per minute was calculated for each parameter. Control values (Control on the graph) were defined as the 1-min mean immediately before the start of drug administration when rACSF was first perfused for 20 min. Regarding the effect of the study drug, the maximum effect was defined as the lowest 1-min mean amplitude after the start of drug administration. The maximum effective values were amplitude, frequency, and duration. Because the primary objective of this study was to discuss the variation in burst amplitude according to burst duration and frequency, the values when the burst amplitude exhibited the maximum effect were considered to be the maximum effective values. Because XII is a secondary neuron of preBötC, it exhibits similar changes to the variation in the preBötC waveform in a healthy state. However, because XII is a different neuron population, a similar relationship in the pathological condition might not be observed. Therefore, the maximum effective values of XII were only selected for the values of XII without considering the maximum effective values of preBötC and their timing. Mean values for the last one minute of drug administration (Pend in the graph) and the last one minute of wash out (Last in the graph) were calculated. Statistical comparison was performed using one-way analysis of variance (one-way ANOVA), Bonferroni analysis for post hoc testing, F analysis, paired *t* test and unpaired *t* test. The significance level was defined as a *p* value < 0.05. Microsoft Office professional plus 2016 (Microsoft corporation, Washington, U.S.) was used for analysis.
